# Positive Psychology Interventions Addressing Pleasure, Engagement, Meaning, Positive Relationships, and Accomplishment Increase Well-Being and Ameliorate Depressive Symptoms: A Randomized, Placebo-Controlled Online Study

**DOI:** 10.3389/fpsyg.2016.00686

**Published:** 2016-05-20

**Authors:** Fabian Gander, René T. Proyer, Willibald Ruch

**Affiliations:** ^1^Department of Psychology, University of ZurichZurich, Switzerland; ^2^Department of Psychology, Martin-Luther University of Halle-WittenbergHalle, Germany

**Keywords:** online intervention, orientations to happiness, positive interventions, positive psychology, PERMA, Well-Being Theory

## Abstract

Seligman (2002) suggested three paths to well-being, the pursuit of pleasure, the pursuit of meaning, and the pursuit of *engagement*, later adding two more, *positive relationships* and *accomplishment*, in his 2011 version. The contribution of these new components to well-being has yet to be addressed. In an online positive psychology intervention study, we randomly assigned 1624 adults aged 18–78 (*M* = 46.13; 79.2% women) to seven conditions. Participants wrote down three things they related to either one of the five components of Seligman's Well-Being theory (Conditions 1–5), all of the five components (Condition 6) or early childhood memories (placebo control condition). We assessed happiness (AHI) and depression (CES-D) before and after the intervention, and 1-, 3-, and 6 months afterwards. Additionally, we considered moderation effects of well-being levels at baseline. Results confirmed that all interventions were effective in increasing happiness and most ameliorated depressive symptoms. The interventions worked best for those in the middle-range of the well-being continuum. We conclude that interventions based on pleasure, engagement, meaning, positive relationships, and accomplishment are effective strategies for increasing well-being and ameliorating depressive symptoms and that positive psychology interventions are most effective for those people in the middle range of the well-being continuum.

## Introduction

Although current psychology mainly focuses on pathologies and attempts to relieve or cure mental illnesses or other malfunctions (see e.g., Myers, 2000; Ruch and Proyer, 2011), it has historically had at least two other objectives: Studying and nurturing talent, and examining ways to make “relatively untroubled” people happier (Seligman et al., 2004; p. 1379). One of the core ideas of Positive Psychology is to strengthen these objectives. *Positive psychology interventions* (PPIs), are interventions that have been developed within Positive Psychology that aim at creating positive outcomes. They have been defined as “[…] treatment methods or intentional activities that aim to cultivate positive feelings, behaviors, or cognitions” (Sin and Lyubomirsky, 2009; p. 468). As such, they are strategies for people who desire to increase their well-being. While most of the research on PPIs has been conducted with non-clinical samples, there are also examples of applications with patients (e.g., Seligman et al., 2006; Casellas-Grau et al., 2014; Huffman et al., 2014). In recent years, research and interest in practical applications of such interventions have steadily increased. Two independent meta-analyses (Sin and Lyubomirsky, 2009; Bolier et al., 2013b) covering a total of 69 randomized controlled studies provide support for the effectiveness of PPIs (increases in subjective and psychological well-being, and amelioration of depression).

The interventions that have been developed and evaluated thus far focus on a broad variety of psychological constructs (e.g., gratitude, hope, goal-attainment, compassion, humor, etc.) and use diverse techniques. Also, most of them do not refer explicitly to a theoretical framework or aim at directly targeting different components of a well-being theory. Interventions that are developed based on a theory of well-being have the advantage that hypotheses can directly be derived from theory and findings can be interpreted within the theory (Lewin, 1951; Michie et al., 2007). Furthermore, they allow for a comparison of the efficacy of different well-being components when used in an intervention (i.e., some well-being components may be more susceptible to change than others). From a more practical perspective, addressing different aspects of well-being based on an individual's well-being profile and their preferences may increase the “fit” between the person and the activity and therefore the effectiveness of an intervention (e.g., Schueller, 2012; Senf and Liau, 2013; Proyer et al., 2015a,b).

In psychology and adjacent disciplines, several conceptualizations of well-being have been proposed (e.g., Ryan and Deci, 2001; Keyes et al., 2002; Seligman, 2002, 2011—to name but a few). Whereas there is no agreement on the number and type of factors that well-being consists of, most theories cover several components of subjective well-being (i.e., cognitive or affective appraisals, such as life satisfaction, positive emotions, or happiness) and psychological well-being (i.e., indicators of optimal functioning, such as having a sense of meaning, or mastery). Seligman (2002) described three basic *orientations* that lead to well-being in his *Authentic Happiness Theory*: The *life of pleasure (P)*, a hedonic orientation that focuses on the experience of positive emotions (a subjective well-being component); the *life of meaning (M)*, a eudaimonic orientation that emphasizes serving a greater purpose; and the *life of engagement (E)*, which focuses on the pursuit of highly engaging and absorbing activities and the thereby elicited experience of *flow* (both could be classified as psychological well-being components). Peterson et al. (2005) developed the *Orientations to Happiness* (OTH) questionnaire to assess the endorsement of these three orientations. Research on the Authentic Happiness model using the OTH revealed that, as expected, all three orientations are positively correlated but not fully overlapping and show robust relations with different indicators of well-being across different cultures (e.g., Peterson et al., 2005; Vella-Brodrick et al., 2009; Chen, 2010; Ruch et al., 2010a; Buschor et al., 2013).

Although the *Authentic Happiness Theory* has been widely quoted in the literature and the OTH is frequently used in research (e.g., Berthold and Ruch, 2014; Ruch et al., 2014; Von Culin et al., 2014; Pollock et al., 2015), Seligman (2011) proposed a revision, the *Well-Being Theory*, which comprises five elements of well-being. To P, E, and M, Seligman added: *Positive relationships* (R) and *accomplishment* (A). Gander et al. (2016) developed short scales for the assessment of R and A using the same framework as the OTH. They report that R and A have minor correlations with each other, P, E, and M, and that they explain additional variance in life satisfaction and flourishing above and beyond the OTH-scales.

It has been argued that few positive psychology studies are tied to a specific model. One example is Giannopoulos and Vella-Brodrick (2011), who used Seligman's (2002) *Authentic Happiness Theory* as a theoretical framework. They compared interventions on the three components of the model regarding their effectiveness in enhancing well-being. Giannopoulos and Vella-Brodrick randomly assigned 218 participants to one of six conditions, instructing them to write down three things related to either: pleasure (1), engagement (2), meaning (3), each of the three orientations (4), three events of the day just passed (5– placebo control condition), or a waitlist control condition (6). All exercises (1–5) were conducted on a daily basis for 1 week. Participants completed a well-being measure (the *Mental Health Continuum—Short Form*, MHC-SF; a composite measure of emotional, social, and psychological well-being; Lamers et al., 2011) before the intervention, after the intervention, and 2 weeks after the intervention. The authors report that well-being increased in all intervention conditions in comparison to the control conditions.

Additionally, Giannopoulos and Vella-Brodrick (2011) examined whether a “fit” between the intervention condition and the participants' baseline scores in the OTH (each scale split into three groups of equal size) would moderate intervention effectiveness. They found some moderation effects that did not yield a consistent pattern: Participants with high baseline scores in pleasure and engagement benefited more when they were assigned to non-matching exercises; namely, when assigned to the engagement or meaning conditions (for pleasure), or the pleasure or meaning conditions (for engagement). The authors did not find any moderating effects for meaning. Thus, this study showed that these orientations can be directly addressed in an intervention and provided first empirical evidence for a potential causal relation of pleasure, engagement, and meaning with well-being.

Gander et al. (2016) conducted the first pilot intervention study based on the five components of Seligman's (2011) Well-Being Theory. They randomly assigned 51 participants to either an intervention condition (IC) or a placebo control condition (describing a route participants had to take this day; e.g., from home to work). Participants in the IC were instructed to write down their experiences on that day that were related to pleasure, engagement, meaning, positive relationships, and accomplishment. Both interventions were conducted daily for 1 week. Before the intervention, after the intervention, 2 weeks and 4 weeks after the intervention, participants completed the OTH, two short scales for the assessment of positive relationships and accomplishment (Gander et al., 2016), and measures of life satisfaction (SWLS; Diener et al., 1985), and affectivity (PANAS; Watson et al., 1988). The authors reported an increase in subjective well-being (i.e., increases in life satisfaction and positive affect, and a decrease in negative affect) in the intervention condition in comparison with the placebo control condition for up to 4 weeks. Additionally, they found an increase in all five well-being components in the intervention condition, while the control condition only showed an increase in R. This preliminary study provided first evidence that the five well-being components can jointly be addressed in an intervention, and that this may be associated with an increase in these components, and in life satisfaction. However, no study has as yet directly *compared* the potential of interventions based on the individual components of Seligman's (2011) Well-Being Theory or addressed the positive associations of accomplishment and positive relationships with well-being in an experimental design. We aim at narrowing this gap by comparing the effects of interventions based on P, E, M, R, and A on well-being in a placebo-controlled study.

The present study has two main aims: (1) Replicating the findings of Giannopoulos and Vella-Brodrick (2011) on the effectiveness of interventions based on pleasure, engagement, and meaning and (2) extending these findings using interventions based on positive relationships and accomplishment. For this purpose, we conducted a randomized, placebo-controlled online-intervention study. In accordance with Giannopoulos and Vella-Brodrick (2011), we adapted the “three good things”-exercise (i.e., writing down three things that went well on that day and reflecting why these things happened; Seligman et al., 2005) by changing the focus of the exercise to experiences related to pleasure, engagement, and meaning. Additionally, we included conditions that address positive relationships and accomplishment. The goal of these interventions was to strengthen the focus on these components in daily life. Table 1 gives an overview on the conditions and the instructions.

**Table 1:** **Descriptions of the six intervention conditions and the placebo control condition**.

**Label**	**Condition**	**Instruction**
		Please take 10 min on every evening for a week before going to bed.
IC1	Pleasure	Remember three things you have experienced today that were related to fun, amusement, joy, or pleasure. Write these three things down and describe how you felt.
IC2	Engagement	Remember three things you have experienced today where your attention was particularly focused and you were not aware of your surroundings. Write these three things down and describe how you felt.
IC3	Positive Relationships	Remember three things you have experienced today that were positive experiences with other people. Write these three things down and describe how you felt.
IC4	Meaning	Remember three things you have experienced today that were personally significant and meaningful. Write these three things down and describe how you felt.
IC5	Accomplishment	Remember three things you have experienced today where you were successful or where you had the impression that you did something really well. Write these three things down and describe how you felt.
IC6	PERMA	Remember one thing from each of the following topics: • Pleasure: something you have experienced on that day that was related to fun, amusement, joy, or pleasure. • Engagement: something you have experienced on that day where your attention was particularly focused and you were not aware of your surroundings • Meaning: something you have experienced on that day that was personally significant and meaningful. • Positive relationships: something you have experienced on that day that was a positive experience with other people. • Accomplishment: something you have experienced on that day where you were successful or where you had the impression that you did something really well. Write these five things down and describe how you felt.
PCC	Early memories	Remember one early childhood memory and write down this memory as detailed as possible.

*IC, Intervention condition. PCC, Placebo control condition*.

In comparison with the study of Giannopoulos and Vella-Brodrick (2011), we changed the design in some respects: Due to the addition of positive relationships and accomplishment, participants in the combination condition were instructed to write down one experience for each of the five components. Furthermore, we used a different placebo control condition, namely the “early memories”-exercise (Seligman et al., 2005). We decided for this condition since the exercise has been proved suitable in numerous intervention studies (i.e., small or no changes in well-being; see Seligman et al., 2005; Gander et al., 2013; Proyer et al., 2015a, Proyer et al., Submitted). Additionally, we considered changes in depressive symptoms, and we asked the participants whether they liked the interventions and whether they perceived a personal benefit from conducting them. This was done to assess indicators of a person × intervention-fit, which was found to be important for long-term effects of such interventions (Proyer et al., 2015b). In line with Giannopoulos and Vella-Brodrick (2011), we also examined possible moderation effects of pleasure, engagement, and meaning (using the OTH; Peterson et al., 2005) and extended their findings by also considering effects of positive relationships and accomplishment (using the measures developed by Gander et al., 2016). Finally, no study has thus far examined the efficacy of positive psychology interventions according to the participant's place on the well-being continuum (Seligman et al., 2004). Therefore, we also tested for moderating effects of baseline levels in happiness and depressive symptoms.

We expected an increase in happiness and a decrease in depressive symptoms in all intervention conditions, in comparison to the placebo control condition. We further expected that participants in the intervention conditions would report higher levels of subjective benefit than those in the placebo control condition. Additionally, we expected differences among the interventions regarding whether participants liked them. We assumed that the interventions targeting P and R would be better liked than the other interventions since they might be closer to the daily experiences of most participants and therefore easier to recall and describe on a daily basis. Since this study is the first to examine interventions based on, as well as moderation effects of, positive relationships and accomplishment, and because Giannopoulos and Vella-Brodrick (2011) did not find a clear pattern of moderation effects, we considered these analyses to be of a more exploratory nature and refrained from stating hypotheses on these analyses. Finally, we expected that the interventions would be more effective for people in the middle range of the well-being spectrum than for those in the lower and upper ranges. This hypothesis was based on the assumption that self-administered interventions would be less appropriate for people with very low scores in well-being, whereas for those with very high scores, there might be ceiling effects in the sense of only little “room for improvement.”

## Methods

### Participants

**Figure 1:**
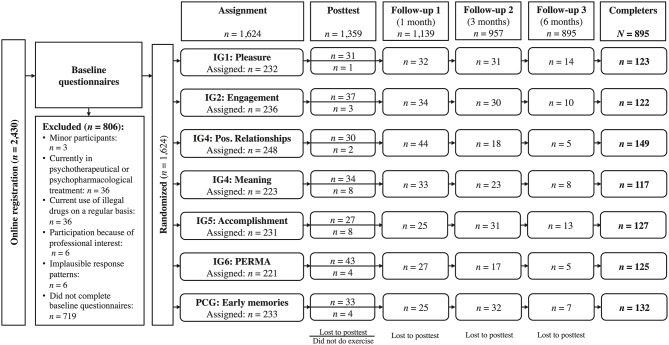
**Flow of participants through each stage of the study**.

We estimated that sample sizes of ≥100 participants per condition would be needed to detect small effects with a power of ≥0.80. However, since large dropout rates were to be expected for the follow-up after 6 months (see Bolier et al., 2013b), we aimed at having sample sizes ≥200 for each of the interventions tested. Participants were recruited through a university press release on a recently published study in various newspapers and magazines in the German speaking countries (unrelated to the content of this study) that included a call for participation in a training program, advertised as a positive psychology program. The study was advertised as a training program for strengths (“strengthen your strengths”) and other positively valued traits. Of the 2430 participants who registered online, 1624 met the inclusion criteria completed the baseline assessments, and were assigned to the intervention condition or the placebo control condition. Inclusion criteria were: Being at least 18 years of age, currently not undergoing psychotherapeutic or psychopharmacologic treatment, no consumption of illegal drugs, not being interested in participating for professional reasons (for preventing biased results), and giving informed consent. The final sample consisted of *N* = 1359 participants (i.e., 83.7% of those who met the inclusion criteria) who completed the assigned intervention. The flow of participants through each stage of the study is shown in Figure 1.

Most participants were female (79.2%) and were of German (75.0%), Swiss (11.4%), or Austrian (10.3%) nationality. Ages ranged from 18 to 78 (*M* = 46.13, *SD* = 11.74). About half of the sample was married or living in a registered partnership (47.1%); about one-fifth of the sample was in a relationship (20.8%, not married or in a registered partnership); about one-fifth of the sample (19.1%) was single, 11.0%, divorced or living in separation; and 2.0% were widowed. More than one-third of the sample (38.3%) had children. The sample was well-educated: 60.9% had a degree from a university or a university of applied sciences, 18.4% had a diploma allowing them to attend a university or university of applied sciences, and 20.7% had completed vocational training or secondary school as their highest education. Most of the participants (74.2%) were currently working; 4.3% were currently unemployed; 7.7% were retired; and the remaining 13.8% were students, interns, apprentices, on holiday, on leave, or neglected to answer the question.

We examined whether those who conducted the assigned exercise differed from those who did not in order to gain information on the generalizability of the findings. Results showed that participants in the latter group were on average 3.1 years younger, *t*_(1622)_ = 3.70, *p* < 0.001 (*d* = 0.26), and tended to be more often men (17.1%) than women (13.3%), $\chi_{\left(1,\;\text{N}\;=\;1622\right)}^{2}$ = 3.41, *p* = 0.07. Whereas there was no difference regarding happiness, *t*_(1622)_ = 1.52, *p* = 0.13, those participants who dropped out of the study tended to report more depressive symptoms, *t*_(1622)_ = 1.89, *p* = 0.06.

In a second step, we analyzed whether randomization was successful by testing for differences among the conditions in demographics and baseline levels of happiness and depressive symptoms: There were no differences among the conditions regarding age, *F*_(6, 1352)_ = 0.75, *p* = 0.61, relationship status, $\chi_{\left(24,\;\text{N}\;=\;1359\right)}^{2}$ = 26.96, *p* = 0.31, education, $\chi_{\left(12,\;\text{N}\;=\;1359\right)}^{2}$ = 15.98, *p* = 0.19, or gender ratio among the conditions, $\chi_{\left(6,\;\text{N}\;=\;1359\right)}^{2}$ = 9.06, *p* = 0.17). However, the conditions differed regarding the participants' working status, $\chi_{\left(18,\;\text{N}\;=\;1359\right)}^{2}$ = 39.38, *p* = 0.003: In the accomplishment condition, more participants were retired (12.8%) than on average (7.7%), in the pleasure condition more participants were unemployed (9.5%), and in the PERMA-condition fewer participants were unemployed (0.6%) than on average (4.3%). However, since the intervention conditions did not—generally—differ from the placebo control condition, we decided not to control for these differences in subsequent analyses. Finally, the conditions did not differ in their baseline scores for happiness, *F*_(6, 1352)_ = 1.65, *p* = 0.13, or for depressive symptoms, *F*_(6, 1352)_ = 1.24, *p* = 28.

### Instruments

The *Authentic Happiness Inventory* (AHI, Seligman et al., 2005; used in a German version as used by Proyer et al., Submitted) is a self-report measurement for the assessment of global happiness and comprises aspects of subjective and psychological well-being that was especially designed for use in intervention studies. The AHI consists of 24 sets of five statements from which the participant has to choose the statement that describes his feelings during the past week best. A sample set of statements ranges from 1 = “*I have sorrow in my life*” to 5 = “*My life is filled with joy*.” Proyer et al. (Submitted) report good psychometric properties for the AHI and showed that it is sensitive to changes in well-being and also covers the top-end of the well-being continuum. The AHI has been often used in research (e.g., Ruch et al., 2010b; Schiffrin and Nelson, 2010; Schueller and Seligman, 2010; Shapira and Mongrain, 2010). The internal consistency in the present study at pretest was high at all measurement time points, ranging from α = 0.94 to α = 0.95.

The *Center for Epidemiologic Studies Depression Scale* (ADS, Radloff, 1977; in the German adaptation by Hautzinger and Bailer, 1993) is a 20-item self-report measurement for the assessment of the frequency and intensity of depressive symptoms in the past week. All items use a 4-point Likert-style scale ranging from 0 [“*rarely or none of the time* (<*1 day*)”] to 3 [“*most or all of the time* (*5–7 days*)”], and four of the 20 items are negatively keyed. A sample item is “I thought my life had been a failure.” The CES-D is one of the most frequently used depression measures and was evaluated as a very balanced and representative measure in a meta-analysis that compared different widely used depression measures (Shafer, 2006). The internal consistency in the present study at pretest was high at all measurement time points, ranging from α = 0.90 to α = 0.92.

The *Orientations to Happiness Questionnaire* (OTH; Peterson et al., 2005; in the German adaption by Ruch et al., 2010a) is an 18-item self-report measurement for the assessment of the three orientations *pleasure, engagement*, and *meaning*, as proposed by Seligman's (2002) Authentic Happiness Theory. All items are positively keyed and are rated on a 5-point Likert-style from 1 (“*very much unlike me*”) to 5 (“*very much like me*”). A sample item is “Life is too short to postpone the pleasures it can provide” (pleasure). Various studies have used the OTH and provided information on its reliability and validity (e.g., Park et al, 2009; Vella-Brodrick et al., 2009; Ruch et al., 2010a). As recommended by Gander et al. (2016) when using the OTH together with the scales for positive relationships and accomplishment, we shortened each scale by one item to reduce the overlap with positive relationships. In the present study, internal consistencies at pretest were acceptable and comparable to earlier findings (pleasure: α = 0.70, engagement: α = 0.66, meaning: α = 0.76).

The *Positive Relationships*- and the *Accomplishment*-scale (Gander et al., 2016) are self-report scales for the assessment of positive relationships and accomplishment consisting of five items each. Together with pleasure, engagement, and meaning, they allow for an assessment of the endorsement toward all components of Seligman's (2011) Well-Being Theory. All items are positively keyed and are rated on the same 5-point Likert-style scale as the OTH. A sample item is “Most things I do give me a feeling of accomplishment.” Gander et al. (2016) reported good factorial validity for the scales when used individually or together with the OTH. They also showed that the scales are able to predict additional variance in life satisfaction and flourishing over and above the influence of the OTH and have high test-retest reliabilities over time periods of 1-, 3-, and 6 months (*r* = 0.68–0.71). In the present study, internal consistencies at pretest were satisfactory (positive relationships: α = 0.75, accomplishment: α = 0.71).

Additionally, upon completing the exercise participants reported their liking of the exercise (from 1 = “*not at all*” to 7 = “*very much*”), whether they saw a personal benefit from the exercise, and how high they perceived this benefit to be (from 1 = “not at all” to 7 = “very high”).

### Procedure

The federal ethics committee of the canton of Zurich, Switzerland provided approval. The whole study was conducted via an online platform (http://www.staerkentraining.ch), which was especially designed for the administration of online intervention studies and was created in accordance with the standards for Internet-delivered testing (Coyne and Bartram, 2006). Participants could register online after completing a form confirming that they fulfilled the inclusion criteria and giving informed consent. After registration they received an individual password which granted them access to the online platform where they were asked to complete basic demographic questionnaires and were again asked whether they fulfill the inclusion criteria. Those participants who failed to do so were excluded from all further analyses; see Figure 1 for details.

Upon completion of the questionnaires, participants could begin the program as soon as they indicated that they would have time to complete the exercise over the following week time. Before participants received their exercise, they had to complete the pretest measures of the AHI, the CES-D, the OTH-scales, and the scales on positive relationships and accomplishment. Those who did not complete the pretest measures or showed an implausible answering pattern (i.e., checking the same answer for every question) were excluded from the analyses. Afterwards, the remaining participants were randomly assigned to one of the six intervention conditions or the placebo control condition via an automated algorithm (based on a Mersenne-Twister) and received their assigned exercise, which had to be conducted every day for 1 week. After the intervention week and one, three, and 6 months later, participants received an e-mail reminder to return to the online platform to complete the posttest and follow-up assessments. At posttest, the participants were asked a “manipulation check” question (i.e., “Did you conduct the assigned exercise?”); only those participants who indicated that they did so entered the following analyses. Finally, participants received automated, individualized feedback on their questionnaires (i.e., on the AHI, CES-D, OTH, and the Positive Relationships- and Accomplishment-scales) after they had completed the last follow-up, but no other incentive for participation was offered. The participants could contact the researchers via e-mail for technical support but no information or help with the exercises was provided.

### Data analysis

All available data was analyzed for participants who completed the assigned exercise. For the evaluation of intervention effectiveness, we applied multi-level modeling using restricted maximum likelihood estimation (REML). This approach offers the advantage that not only those participants who completed all follow-ups enter the analysis (as in a classical repeated measurement ANOVA approach) but also the available data from participants who have missing data at some time points (cf. Singer and Willett, 2003). To estimate the overall intervention effectiveness, we analyzed the effect of “condition” (each intervention condition separately vs. the placebo control condition) on all measurement time points for happiness and depressive symptoms after the intervention week, while controlling for the pretest scores of happiness and depressive symptoms, respectively. The model was as follows:

$$\begin{array}{l} Y_{i,\;j}=\gamma_{00}+\gamma_{01}Baseline_{i}+\gamma_{02}Condition_{i}+\left(\varepsilon_{ij}+\zeta_{0i}\right) \end{array}$$

where

$$\begin{array}{l} \varepsilon_{ij}\sim N\left(0,\sigma_{\varepsilon }^{2}\right)\;and\;\zeta_{0i}\sim N\left(0,\sigma_{0}^{2}\right) \end{array}$$

For the estimation of the overall effectiveness *Y*_*ij*_ refers to the scores of happiness or depression at all measurement time points after the intervention, *Baseline* refers to the pre-test scores in happiness or depression, and *Condition* refers to the intervention condition of interest (coded as 1) vs. the placebo control condition (coded as 0).

Subsequently, we analyzed the effect of “condition” on each assessment of happiness and depressive symptoms after the intervention separately, again controlling for the pretest scores. For these analyses, *Y*_*ij*_ refers to the scores of happiness or depression at the time point of interest. Unless otherwise indicated, always the fixed effect of “condition” is given.

For the analysis of moderating effects we computed tertile splits for the pleasure-, engagement-, meaning-, positive relationships-, and accomplishment-scales (low, medium, and high scorers), in accordance with Giannopoulos and Vella-Brodrick (2011). Using the moderator variable and its interaction with the condition as additional predictors, we conducted the same analyses of overall effects as described above. When these analyses revealed a significant interaction between a moderator and the condition, we computed simple main effects. These were done to compare the increases in happiness and depressive symptoms between each intervention condition and the placebo control condition separately for low-, medium-, and high-scorers in each orientation. For the analysis of moderating effects of baseline happiness and depressive symptoms, we conducted the same analyses of overall effects but also included the interaction of the baseline scores with the condition, the squared baseline scores, and their interaction with the condition to test whether the baseline scores might have non-linear effects on the outcomes (e.g., stronger effects for those in the medium range of the happiness scale).

We based our analyses and interpretation of the findings on those participants who conducted the assigned exercise because this allows for determining whether the interventions are effective in increasing happiness in those who conducted the interventions. However, one might argue that dropping out is systematically related to an individual difference variable (such as age; see below), possibly indicating that the interventions might not have been effective for these participants. From a more applied perspective, it is also of interest whether those the interventions would still be effective if the participants who dropped out (regardless of whether they conducted the interventions or not) would also be considered. For this purpose, we repeated the above-mentioned analyses in an intention-to-treat (ITT)-approach, using all participants that were assigned to the conditions and estimating all missing data points. These estimations were obtained using the Multivariate Imputation by Chained Equations (MICE) method in which “[…] a series of regression models are run whereby each variable with missing data is modeled conditional upon the other variables in the data” (Azur et al., 2011; p. 2). This procedure is repeated to generate multiple imputed dataset (we used 20 imputed datasets). These datasets are then analyzed separately and the results are pooled to obtain an overall estimation. We used the R-package “mice” (van Buuren and Groothuis-Oudshoorn, 2011) for this procedure. The raw data are provided as an online supplementary material (Data Sheet 1).

## Results

### Intervention effectiveness

The means and standard deviations can be found in Table 2, whereas the results of the multilevel analyses for intervention effectiveness are given in Table 3 (fixed effects for the condition are given in the prediction of happiness or depressive symptoms after the intervention while controlling for the baseline levels in happiness and depressive symptoms).

**Table 2:** **Means and standard deviations of the 10 groups at the five time periods for happiness and depressive symptoms**.

	**Pre**			**Post**			**1 M**			**3 M**			**6 M**		
	***N***	***M***	***SD***	***N***	***M***	***SD***	***N***	***M***	***SD***	***N***	***M***	***SD***	***N***	***M***	***SD***
**HAPPINESS**															
P	200	2.94	0.6	200	3.02	0.57	169	3.09	0.59	142	3.12	0.56	144	3.21	0.53
E	196	2.95	0.59	196	3.04	0.58	164	3.09	0.59	142	3.12	0.57	150	3.14	0.61
R	216	2.95	0.57	216	3.02	0.61	174	3.05	0.61	163	3.11	0.62	167	3.15	0.6
M	181	3.03	0.5	181	3.1	0.53	150	3.18	0.54	130	3.16	0.56	138	3.2	0.57
A	196	2.87	0.6	196	2.95	0.6	172	2.99	0.61	147	3.03	0.59	150	3.1	0.62
PERMA	174	3.02	0.54	174	3.08	0.5	148	3.13	0.55	133	3.11	0.56	143	3.17	0.52
PCG	196	2.99	0.56	196	3	0.56	171	3.05	0.62	150	3.02	0.57	149	3.12	0.58
**DEPRESSIVE SYMPTOMS**															
P	200	0.69	0.45	200	0.6	0.43	168	0.61	0.48	141	0.58	0.45	143	0.56	0.42
E	196	0.71	0.47	196	0.6	0.41	162	0.6	0.44	141	0.62	0.44	150	0.62	0.49
R	216	0.71	0.45	216	0.62	0.44	172	0.58	0.43	163	0.58	0.46	166	0.57	0.45
M	181	0.66	0.41	181	0.6	0.43	148	0.55	0.4	129	0.58	0.42	138	0.58	0.44
A	196	0.75	0.49	196	0.64	0.44	171	0.68	0.5	145	0.66	0.46	148	0.62	0.47
PERMA	174	0.64	0.42	174	0.57	0.4	147	0.55	0.44	133	0.63	0.51	141	0.56	0.42
PCG	196	0.71	0.46	196	0.67	0.44	171	0.67	0.46	148	0.68	0.45	149	0.64	0.49

*Happiness, Authentic Happiness Inventory; Depression, Center for Epidemiologic Studies Depression Scale; PCG, Early memories; 1 M, one month after the intervention; 3 M, three months after the intervention; 6 M, six months after the intervention*.

**Table 3:** **Fixed effects of the experimental condition for happiness and depressive symptoms**.

	**Overall**			**Post**			**1 M**			**3 M**			**6 M**		
	***df***	***F***	***f*^2^**	***df***	***F***	***f*^2^**	***df***	***F***	***f*^2^**	***df***	***F***	***f*^2^**	***df***	***F***	***f*^2^**
**HAPPINESS**															
P	1, 393	5.91^**^	0.013	1, 393	4.74^*^	0.009	1, 337	2.42^†^	0.004	1, 289	5.91^*^	0.017	1, 290	3.46^*^	0.008
E	1, 389	5.54^**^	0.018	1, 389	6.48^**^	0.014	1, 332	2.86^*^	0.006	1, 289	5.37^*^	0.015	1, 296	1.06	0
R	1, 409	4.88^*^	0.016	1, 409	3.47^*^	0.006	1, 342	2.40^†^	0.004	1, 310	7.43^**^	0.02	1, 313	1.77^†^	0.002
M	1, 374	5.52^**^	0.017	1, 374	5.13^*^	0.011	1, 318	5.98^*^	0.015	1, 277	4.04^*^	0.011	1, 284	0.54	0
A	1, 389	2.98^*^	0.008	1, 389	3.62^*^	0.007	1, 340	0.54	0	1, 294	4.49^*^	0.012	1, 296	2.90^*^	0.006
PERMA	1, 367	3.59^*^	0.01	1, 367	3.25^*^	0.006	1, 316	2.40	0.004	1, 280	3.69^*^	0.009	1, 289	0.49	0
**DEPRESSIVE SYMPTOMS**															
P	1, 393	3.19^*^	0.014	1, 393	3.38^*^	0.006	1, 336	0.68	0	1, 286	2.94^*^	0.007	1, 289	1.46	0.001
E	1, 389	3.14^*^	0.011	1, 389	5.16^*^	0.011	1, 330	1.53	0	1, 286	1.06	0	1, 296	0.26	0
R	1, 409	8.22^**^	0.036	1, 409	3.14^*^	0.005	1, 340	7.08^**^	0.018	1, 308	5.15^*^	0.013	1, 312	3.63^*^	0.008
M	1, 374	2.79^*^	0.01	1, 374	1.14	0	1, 316	3.75^*^	0.009	1, 274	1.80^†^	0.003	1, 284	0.35	0
A	1, 389	1.41	0.001	1, 389	2.95^*^	0.005	1, 339	0.14	0.002	1, 290	0.81	0	1, 294	2.78^*^	0.006
PERMA	1, 368	3.66^*^	0.014	1, 367	2.67	0.005	1, 315	4.00^*^	0.009	1, 278	0.35	0.003	1, 287	1.09	0

*Sample sizes are given in Table 2. Happiness, Authentic Happiness Inventory, Depression, Center for Epidemiologic Studies Depression Scale; 1 M, one month after the intervention; 3 M, three months after the intervention; 6 M, six months after the intervention; f^2^, Cohen's f^2^*.

† p < 0.10;

* p < 0.05;

** *p < 0.01 (one-tailed)*.

Table 3 shows that, overall, participants in all intervention conditions reported higher scores in happiness after the intervention than the placebo control condition when controlling for the pretest scores. An inspection of the individual time points revealed that this effect was observable in all conditions at the immediate posttest and at the 3-month follow-up. At the 1-month follow-up, two conditions (i.e., engagement and meaning) scored significantly higher than the placebo control condition and there was a (non-significant) trend in the same direction in two other conditions (i.e., pleasure, and positive relationships). In two conditions (i.e., pleasure, and accomplishment) the positive effects on happiness were observable up to 6 months after the intervention, whereas in the positive relationships condition the effects failed to reach significance.

All interventions led to an amelioration of depressive symptoms across all time points compared to the placebo control condition, except for the accomplishment-condition. Depending on the condition, this reduction in depressive symptoms could be observed up to the immediate post-test (engagement), 1 month (meaning and PERMA), 3 months (pleasure), and 6 months (positive relationships and accomplishment).

### Intention-to-treat analyses

Results for the intention-to-treat analyses using multiply imputed datasets were highly parallel as for the analyses on those participants who did not drop out of the study (fixed effects of condition are given in Table 4). Despite of the effect sizes being smaller in size in general. The interventions led to higher happiness scores in comparison with the placebo control condition when analyzing all time points after the intervention jointly and controlling for the pretest scores. This effect was observable for up to 3 months in all conditions. Overall, most conditions also led to a reduction in depressive symptoms; the exceptions were the engagement and the accomplishment conditions that yielded marginally significant results. For the individual time points, the same results were obtained as for the analyses without the dropouts, the only difference was that those conditions that reduced depressive symptoms after 6 months (i.e., positive relationships and accomplishment) only yielded marginally significant results.

**Table 4:** **Fixed effects of the experimental condition for happiness and depressive symptoms using multiple imputed dataset (ITT)**.

	***N***	***df***	**Overall**		**Post**		**1 M**		**3 M**		**6 M**	
			***F***	***f*^2^**	***F***	***f*^2^**	***F***	***f*^2^**	***F***	***f*^2^**	***F***	***f*^2^**
**HAPPINESS**												
P	232	1, 462	5.91^**^	0.013	4.13^*^	0.008	1.36	0.002	7.62^**^	0.018	2.08^†^	1.005
E	236	1, 467	4.12^*^	0.009	5.37^*^	0.011	1.56	0	4.91^*^	0.013	0.57	0
R	248	1, 478	4.38^*^	0.008	3.35^*^	0.006	0.97	0	7.08^**^	0.015	1.1	0
M	223	1, 454	5.30^*^	0.011	5.79^**^	0.011	4.13^*^	0.009	4.30^*^	0.011	0.79	0
A	231	1, 461	2.94^*^	0.006	3.74^*^	0.007	0.57	0	3.03^*^	0.006	1.63	0
PERMA	221	1, 451	3.05^*^	0.007	3.19^*^	0.006	1.66^†^	0.003	3.23^*^	0.008	0.61	0
**DEPRESSIVE SYMPTOMS**												
P	232	1, 462	3.49^*^	0.008	3.28^*^	0.006	0.75	0	3.49^*^	0.008	0.92	0
E	236	1, 467	2.50^†^	0.006	4.79^*^	0.011	1.34	0	0.99	0	0.25	0
R	248	1, 478	7.80^**^	0.018	4.17^*^	0.008	4.79^*^	0.01	3.72^*^	0.01	2.51^†^	0.005
M	223	1, 454	3.67^*^	0.009	2.10^†^	0.003	3.39^*^	0.008	1.86^†^	0.005	0.52	0
A	231	1, 461	2.28^†^	0.004	3.75^*^	0.007	0.27	0	0.55	0	1.82^†^	0.003
PERMA	221	1, 451	3.13^*^	0.007	2.66^†^	0.005	2.98^*^	0.006	0.54	0	1.05	0.001

*n_Placebo_, 233. Only the effects for condition (each intervention condition vs. placebo control condition) are displayed. Happiness, Authentic Happiness Inventory; Depression, Center for Epidemiologic Studies Depression Scale; 1 M, one month after the intervention; 3 M, three months after the intervention; 6 M, six months after the intervention; f^2^, Cohen's f^2^*.

† p < 0.10;

* p < 0.05;

** *p < 0.01 (one-tailed)*.

### Moderation effects

As a next step, we examined the influence of specific characteristics of the participants on the outcomes (i.e., we explored whether the changes in happiness and depressive symptoms were stronger when the assigned intervention matched the participants' scores on the pleasure-, engagement-, meaning-, the positive relationships-, and the accomplishment-scales). Results showed that the effectiveness of the engagement condition was moderated by pleasure; happiness: *F*_(2, 385)_ = 3.45, *p* = 0.03, *f* ^2^ = 0.018, and depressive symptoms: *F*_(2, 385)_ = 3.88, *p* = 0.02, *f* ^2^ = 0.022. Happiness increased in those with low, *F*_(1, 385)_ = 6.69, *p* = 0.01, or medium levels of pleasure, *F*_(1, 385)_ = 4.86, *p* = 0.03, but not for those with high levels of pleasure, *F*_(1, 385)_ = 0.67, *p* = 0.41. Depressive symptoms decreased mainly in those with medium levels of pleasure, *F*_(1, 385)_ = 6.64, *p* = 0.01, but not in those with low, *F*_(1, 385)_ = 3.02, *p* = 0.08, or high levels, *F*_(1, 385)_ = 1.09, *p* = 0.30. Furthermore, the effectiveness of the meaning condition was moderated by the baseline scores in meaning, *F*_(2, 389)_ = 3.28, *p* = 0.04, *f* ^2^ = 0.017: Happiness increased, *F*_(1, 389)_ = 11.53, *p* = 0.001, and depressive symptoms decreased, *F*_(1, 389)_ = 7.95, *p* = 0.01, for those with high levels of meaning, but not for those with low levels, *F*_(1, 389)_ = 0.34, *p* = 0.56, *F*_(1, 389)_ = 0.81, *p* = 0.37, or medium levels, *F*_(1, 389)_ = 0.04, *p* = 0.84, *F*_(1, 389)_ = 0.72, *p* = 0.40.

Further, we analyzed whether the baseline scores in happiness and depressive symptoms moderated the findings. In order to test for linear and quadratic effects, we included the baseline scores, squared baseline scores, and their interaction with condition as additional predictors. Firstly, we compared the all intervention conditions combined with the placebo control condition to test for a general trend in the data. Secondly, we computed the same analyses separately for the intervention conditions (comparing each with the placebo control condition). Table 5 gives the interaction terms between the condition and the squared baseline scores (quadratic effects).

**Table 5:** **Moderation effects of the baseline AHI and CES-D on happiness and depressive symptoms**.

**Conditions**	***N***	***Df***	**Happiness**		**Depression**	
			***F***	***f*^2^**	***F***	***f*^2^**
All vs. PCG	1359	1, 1353	3.51^*^	0.004	3.23^*^	0.002
P	200	1, 390	2.12^†^	0.004	5.57^**^	0.023
E	196	1, 386	2.41^†^	0.007	0	0
R	216	1, 406	1.19	0.001	2.47^†^	0.003
M	181	1, 371	1.98^†^	0.006	1.92^†^	0.004
A	196	1, 386	1.62	0.002	4.65^*^	0.012
PERMA	174	1, 364	3.45^*^	0.009	3.28^*^	0.002

*n_Placebo_, 196. Only the interactions between squared happiness/depressive symptoms and the condition (all fixed effects) are given. Happiness, Authentic Happiness Inventory, Depression, Center for Epidemiologic Studies Depression Scale, f^2^, Cohen's f^2^*.

† p < 0.10;

* p < 0.05;

** *p < 0.01 (one-tailed)*.

Table 5 shows that for both happiness and depressive symptoms an interaction was found between the conditions and (the square of the) baseline happiness (or depressive symptoms, respectively)—when comparing all intervention conditions with the placebo control condition. When analyzing the intervention conditions separately, quadratic effects (or trends) of the baseline scores for both outcomes, happiness and depression, were found for most intervention conditions.

**Figure 2:**
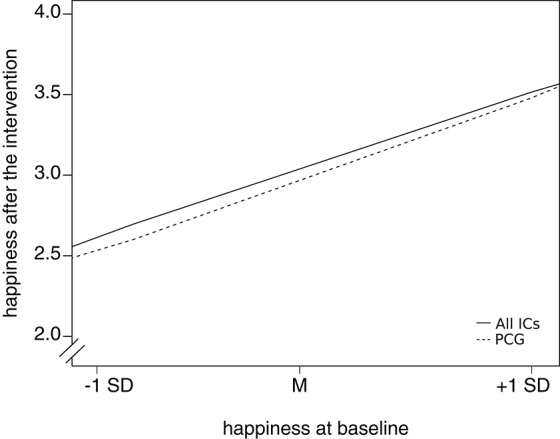
**The relationship between baseline scores in happiness and the happiness scores after the intervention (all measurement time points combined) for all intervention conditions combined (all ICs) vs. the placebo control condition (PCG)**.

A visual inspection of the findings revealed that, as expected, the interventions were most effective for those participants whose baseline scores in happiness and depressive symptoms were average (i.e., ~ ±1 *SD*), or slightly below average for happiness and slightly above average for depressive symptoms. For those participants who had low or high scores at baseline, the intervention was less effective. Figure 2 shows the relationship for this interaction between the baseline scores in happiness and the intervention condition on happiness after the intervention.

### Liking and benefit

We tested whether the conditions differed in terms of how much the participants liked the interventions and whether they perceived a personal benefit from the intervention. ANOVA results indicated that the conditions differed in both aspects, liking: *F*_(6, 1352)_ = 4.92, *p* < 0.001, and benefit: *F*_(6, 1352)_ = 4.19, *p* < 0.001. Means, Standard Deviations, and results of *Post-hoc* tests (LSD) are given in Table 6.

**Table 6:** **Means and standard deviations for liking the interventions and benefiting from them**.

	***N***	**Liking**		**Benefit**	
		***M***	***SD***	***M***	***SD***
P	200	5.71_a_	1.02	3.31_a_	0.77
E	196	5.24_b_	1.12	3.08_bc_	0.69
R	216	5.52_ac_	1.08	3.30_a_	0.83
M	181	5.33_bc_	1.23	3.23_ac_	0.8
A	196	5.28_b_	1.19	3.16_ac_	0.78
PERMA	174	5.52_a_	1.11	3.22_ac_	0.66
PCG	196	5.23_b_	1.2	3.01_b_	0.79

*A shared subscript indicates that these groups did not differ at p < 0.05 (LSD-procedure). Liking: How participants liked the exercise (1, not at all; 7, very much). Benefit: The extent to which participants perceived a benefit from the exercise (1, not at all; 5, very high)*.

Table 6 shows that participants liked the interventions for pleasure, positive relationships, and PERMA better than the placebo control exercise and indicated that they benefited more from most exercises (except for engagement) than from the placebo control condition. Moreover, there were some differences among the interventions: For example, participants indicated greater liking of the pleasure-, positive relationships-, and PERMA-interventions than the engagement- and the accomplishment-interventions. They also reported perceiving more benefit from the pleasure- or the positive relationships-intervention than from the engagement intervention.

## Discussion

This study shows that self-administered online-interventions based on pleasure (P), engagement (E), positive relationships (R), meaning (M), or accomplishment (A) are effective for increasing happiness and alleviating depressive symptoms for up to 6 months. The study confirms earlier findings of Giannopoulos and Vella-Brodrick (2011) and is the first study to directly evaluate interventions based on the five components of Seligman's (2011) Well-Being Theory.

We successfully replicated the findings of Giannopoulos and Vella-Brodrick (2011) on the effectiveness of interventions based on pleasure, engagement, and meaning, and we additionally extended previous knowledge by showing that the effects of these interventions can be found for longer time periods than expected, namely for up to 3 months for engagement and meaning and for up to 6 months for pleasure. Moreover, we also considered changes in depressive symptoms and found the interventions to alleviate depressive symptoms in the short term (engagement), up to 1 month (meaning), or several months later (pleasure).

The findings for the interventions based on positive relationships and accomplishment are encouraging: Both interventions increased happiness for 3 and 6 months but had differential effects on depressive symptoms. Whereas no overall effect for the reduction in depressive symptoms was found in the accomplishment condition (only at the immediate posttest and at the 6-month follow-up), the participants in the positive relationships-condition reported strong decreases in depressive symptoms at every time point and yielded by far the strongest effects of all conditions. The results for the accomplishment-condition should be interpreted with some caution since participants in this condition reported the highest levels of depressive symptoms and the lowest levels of happiness at baseline (although not significantly different from the baseline levels of the other conditions). This might have had an impact on the effectiveness of the intervention. However, this restriction does not apply to the positive relationships-condition, and our results seem to corroborate the notion that “[…] other people are the best antidote to the downs of life” (Seligman, 2011; p. 20). Thus, from an applied perspective, focusing on positive relationship experiences might be a potent strategy for reducing depressive feelings in non-depressed participants, which should be examined in further detail. However, future studies should also examine exactly what participants did (e.g., whether the intervention lead to cognitive changes in the appreciation of one's relationships or also lead to an increase in the time spent with other people) to offer a more thorough insight in the mechanisms of this intervention. Furthermore, information on the size and quality of the participants' social networks should be considered since these factors might be important moderators for the effectiveness of this intervention.

In comparison with earlier studies (Giannopoulos and Vella-Brodrick, 2011), we only found moderation effects for the engagement condition (moderated by the baseline scores in pleasure), and the meaning condition (moderated by the baseline scores in meaning). However, taking the large number of comparisons into account, these effects were relatively small and we have refrained from interpreting them. We also found (trends for) moderation effects of the baseline scores in happiness and depressive symptoms for most interventions. This provides first evidence for a basic idea in positive psychology, namely that these interventions are most effective for those people in the middle range of the well-being continuum and potentially less useful for those who are very depressed or already flourishing. Since meta-analyses reported stronger effects for those participants with elevated scores in depression or those suffering from specific psychosocial problems (Sin and Lyubomirsky, 2009; Bolier et al., 2013b), it might be the case that this finding only applies to self-administered interventions, where the participants' self-regulation might play a more important role than when interventions are overseen by a therapist (see also Proyer et al., 2013).

As in the study by Giannopoulos and Vella-Brodrick (2011) the combined condition that focused on all aspects simultaneously did not outperform the other conditions but showed numerically rather small effects in comparison. It could be argued that this exercise is cognitively different from the other exercises since it fosters a more superficial engagement across different topics, whereas the other exercises focus on one particular topic. Thus, this exercise might also differ with regard to its emotional impact since it could be frustrating if no examples for all of the PERMA domains can be found.

Although we were not able to collect information on exactly what the participants did in the interventions, there is evidence that the tested interventions—despite using the same intervention strategy (i.e., writing down three positive things on a daily basis)—represent different ways of addressing well-being. Firstly, our results suggest that different interventions yield differential results; whereas some interventions have strong and long-lasting effects on the reduction of depressive symptoms (positive relationships), others show small, or no effects at all on depressive symptoms (accomplishment). Secondly, we find differences in the preferences for each intervention since some interventions were better liked (e.g., pleasure vs. engagement) or rated higher in terms of whether participants subjectively benefited from them (e.g., positive relationships vs. engagement). Nonetheless, these findings are indicative of differences among the conditions, which should be examined in more detail in future studies.

Several limitations of this study have to be acknowledged. As with many other studies examining positive psychology interventions (i.e., Mitchell et al., 2009; Lyubomirsky et al., 2011; Bolier et al., 2013a; Gander et al., 2013), the present study did not have a balanced gender-ratio. Although we have no reason to expect gender effects, and no study so far has reported an impact of gender on the effectiveness of positive psychology interventions, it would be worth examining why such positive psychology interventions attract more females than males or whether gender makes a difference when participants are not self-selected. Also, we found that dropout rates decrease with age, whereas Sin and Lyubomirsky (2009) report an increase of intervention effectiveness with age. Both findings could be attributed to the increase of self-regulation with age (e.g., Ruch et al., 2010b). For practical applications it may be helpful to redesign the interventions for younger individuals; for example, by increasing the commitment through having participants to sign an agreement for completing all parts of a program, or sending daily reminders to complete an exercise.

Further, a considerable part of the sample that was assigned an intervention dropped out (i.e., did not complete the posttest or did not complete the assigned exercise; 16.3%), or did not complete all of the follow-ups (44.9%). This is a common problem in online intervention studies, and these rates are rather low in comparison to other studies (cf. Mitchell et al., 2010; Gander et al., 2013). We cannot rule out that our findings are affected by the missing information. However, the highly parallel results for the intention-to-treat analyses suggest that no large differences to the obtained results could be expected had the complete information been available. Finally, it cannot be ruled out that the finding that intervention works best in the middle-range of the well-being continuum is due to measurement issues, such as ceiling or floor effects. However, previous investigations of the scale suggested that this is rather unlikely (see Proyer et al., Submitted).

## Conclusion

The present study revealed three major outcomes: (1) Interventions based on accomplishment and positive relationships are effective strategies for increasing happiness, whereas a positive relationships-based intervention also ameliorated depressive symptoms; (2) the study replicated the findings of Giannopoulos and Vella-Brodrick (2011) and confirmed that interventions based on pleasure, engagement, and meaning are effective in increasing well-being and ameliorating depressive symptoms across different cultural settings and for longer time periods than expected; and (3) provides initial support for the notion that self-administered positive psychology interventions based on the PERMA-model are most effective for those people in the middle range of the well-being continuum.

## Author contributions

All authors were involved in the planning of the study, the data analysis, and the writing of the article. FG conducted the data collection.

## Funding

The preparation of this paper has been facilitated by research grants of the Swiss National Science Foundation (SNSF; 100014_132512 and 100014_149772) awarded to RP and WR.

### Conflict of interest statement

The authors declare that the research was conducted in the absence of any commercial or financial relationships that could be construed as a potential conflict of interest.
